# A pilot summer day camp cooking curriculum to influence family meals

**DOI:** 10.1186/s40814-019-0528-0

**Published:** 2019-12-13

**Authors:** Lindy Williams, Aleshia Magee, Cameron Kilby, Katherine Maxey, Joseph A. Skelton

**Affiliations:** 1 Wake Forest School of Medicine, Winston-Salem, NC USA; 2 YMCA of Northwest North Carolina, Winston-Salem, NC 27157 USA; 3 Department of Pediatrics, Wake Forest School of Medicine, Medical Center Blvd, Winston-Salem, NC USA; 4 Brenner FIT (Families In Training) Program, Brenner Children’s Hospital, Wake Forest Baptist Health, Winston-Salem, NC USA

**Keywords:** Children, Meals, Cooking, Nutrition, Camp

## Abstract

**Background:**

Efforts to combat the epidemic of childhood obesity have approached the issue from many different angles, with a family approach being the gold standard. While most efforts focus on the parents, few have viewed the child as the agent of change. In this study, we explored the feasibility of implementing a cooking curriculum into a summer day camp to determine its reception and explore the potential of home reach.

**Methods:**

In partnership with a local YMCA, a child-focused cooking curriculum was developed, designed to be delivered to various age groups with key nutritional messages. Interviews were conducted with participating children and their parents to determine acceptability and potential to influence the home environment as well as explore children’s understanding of nutrition and cooking topics.

**Results:**

Children in the study ranged from 7 to 15 years of age. Children overwhelmingly enjoyed the cooking camp and talked about it with their parents at home. Almost all parents had plans to try the recipes at home, and many had already made one or more of the recipes.

**Conclusions:**

It is feasible to incorporate cooking lessons into a children’s summer day camp, with some evidence of reach into the home. Future studies should evaluate children as agents of change in cooking and meal preparation, and assess if this could increase the number and quality of family meals.

## Introduction

The prevalence of obesity has more than doubled in children and quadrupled in adolescents over the past 30 years [1]; in children 6–15 years of age, the prevalence of obesity ranges from 18 to 21% [2], with severe obesity continuing to worsen in these age groups [3]. Efforts to address childhood obesity have taken many different angles. One is a school-based approach, including providing healthy meals, educating students about nutrition, and/or encouraging physical activity as steps to prevent obesity [4]. One advantage of this approach is it involves professionals skilled at educating and encouraging youth. It also can be administered on a relatively large scale and incorporate many variables that contribute to childhood obesity (i.e., activity, nutritional intake, and education). Nonetheless, only approximately half of the school-based interventions prove to be successful in preventing obesity or improving the weight status of students [4]. The lack of overwhelming success is likely because school is only one of many factors involved in health habits, and to be more successful, an intervention would need to address other aspects of a child’s life to make a meaningful change.

Family-based behavioral interventions are considered the “gold standard” for the prevention and treatment of pediatric obesity, since they incorporate the vital role parents play in shaping children’s health behaviors [5] both with respect to genes and environment [4, 6, 7]. Furthermore, parents decide what, when, and how much a child is fed for most meals, and they can structure daily schedules to limit sedentary activity while facilitating physical activity. In particular, the frequency of meals eaten together as a family has been proven to correlate with lower rates of obesity [8–10]. Fulkerson et al. featured family meals as a core part of the interventions in the Healthy Home Offerings via the Mealtime Environment (HOME) study, with promising results [11, 12]. This is one of the few studies intervening on the family meal environment and utilizes family-based interventions. In fact, there is a large body of literature that supports parents being the exclusive agent of change in childhood obesity treatment [13–18].

It is important to devise new and innovative methods to address childhood obesity. While many studies have focused on parents as the agents of change, few have been conducted to explore how children can be agents of change in their home environment to advocate for a healthier lifestyle [19]. Research in other areas demonstrates children can instigate change within the family, across topics such as smoking cessation [20–23] and environmental conservation [24–27]. A few preliminary studies show that children also can be agents of change to affect health behaviors within families [28, 29], but this concept has not been explored in great detail. In regard to increasing family meals, children can often be an obstacle to making these changes [30]. Therefore, exploring children as agents of change within the family could be an innovative approach to changing family meal patterns; involving children in meal preparation has shown promise to increase variety and positivity around food, even increasing vegetable intake [31]. This “child-focus” could be a complementary approach to family-based interventions, and is supported by the socio-ecological model of obesity [6], as well as tenets of family systems theory [32], which views the individual (in this scenario, the child) in the context of the family, recognizing the subsystems at play.

Intervening with children in community settings is a practical and efficient approach to improving health, particularly if the family may also be reached. As working with children in cooking and meal preparation has shown promise to change nutrition habits, intervening on children to increase their culinary skills in a community setting is promising; determining if that impact extends to the home environment would be additional outcome of interest. A group of medical students, faculty, and dietitians at Wake Forest School of Medicine designed a child-focused cooking curriculum that was piloted at a local YMCA in Winston-Salem, North Carolina, during their summer day camp in 2016. Recipes for the summer cooking camp were developed by experienced dietitians in Brenner FIT®, a multidisciplinary pediatric weight management program. Recipes were chosen to fit the balanced plate and cover general nutrition topics in an age-appropriate manner. Each recipe was linked to a cooking skill or concept and teaching focus. For example, the recipes for roasted broccoli, caramelized Brussel sprouts, and sautéed asparagus built on the previous week’s lesson of knife skills and stove-top cooking. The teaching focus was introducing new ways to prepare fruits and vegetables (part of the balanced plate) and use of common kitchen appliances and tools. Small groups of 5–6 students were brought to the kitchen daily for a cooking class that summer. The sessions appeared to be enjoyed by the children attending, with many comments by parents attesting to that and requesting copies of the recipes made. Because of the initial positive reception, it was decided by a research team at Wake Forest School of Medicine and staff at the YMCA to repeat the cooking curriculum during the subsequent summer day camp, and determine the feasibility of using the intervention to impact family meals and child knowledge.

The overall objective of this study is to explore the feasibility of children being agents of change in the home cooking environment by them experiencing a cooking curriculum at a summer day camp. We describe the community-engaged development and implementation of the cooking curriculum within an existing summer day camp experience and its potential to influence child and family nutritional habits.

## Methods

### Design

This was an exploratory pilot study, building on a trial program from the previous summer, to (1) determine the acceptability and feasibility of a cooking curriculum within a YMCA summer day camp and (2) explore whether there is the potential for the child-focused program to reach into the home environment, as a means to increase family meals and modify child eating behaviors. The study employed qualitative interviews of children and parents to assess acceptability and potential of extension into the home, as well as qualitative assessment of children’s learning of nutrition and culinary topics. The study was planned in conjunction with the YMCA summer camp leadership using principles of community-engaged research [33], including YMCA staff in research planning and execution of the study, as well as modification of the cooking curriculum.

### Participants

The cooking camp curriculum was piloted with children enrolled in a summer day camp at a local YMCA in Winston-Salem, NC, in 2017. Parents and children ages 7–15 years of age attending summer day camp sessions at the YMCA were eligible to participate. In total, three hundred twenty-six different children attended the day camp. Of those, 195 (60%) were on financial assistance. On average, there were 119 children at camp each day; 48% of participants were Caucasian, 40% African American, 7% Hispanic, and 5% of Asian descent. All participants were from within the city limits of Winston-Salem, North Carolina. Nearly all of the children were able to participate in at least one cooking class. Interview participants were recruited from this summer day camp population.

### Procedures

Children and parents were recruited to participate in qualitative interviews from the day camp population described above. Upon starting camp at the beginning of summer, parents of campers were informed of the cooking classes and research study via emails and newsletters (see ethical approval). Five to six weeks into the camp, we began qualitative interviews of campers and parents. Campers were selected randomly at the end of their cooking lessons and asked to participate in the study. Parents were asked during “ride out” (area where parents would let children out of the car or pick them up) if they would be willing to participate via telephone interview. Those who agreed were later called for the structured interviews. Participation was completely voluntary; no incentives were provided to participate. The interviews aimed to capture children’s perceptions and understanding of nutrition and cooking topics. Two study staff members participated in each interview; one led the interview, asking questions and recording responses, and the other wrote down participant responses. After each interview, the study staff compared notes and responses and recorded general impressions of the interview.

The research team used principles of community-engaged research, a collaborative process between researchers and community members, in this study [34]. This approach was used to design and adapt the research plan to the YMCA setting, and give YMCA staff a voice in the process, and position the program for sustainability and dissemination. Key leaders in the YMCA summer day camp participated in all aspects of this project, especially in the planning of data collection and interpretation, first in the design of the cooking camp curriculum, particularly in the pacing of classes and scheduling during the camp day.

The initial trial of the cooking curriculum the previous summer was with older children (> 10 years) and in smaller numbers (groups of 5–6 who requested to attend). The YMCA requested expansion of the cooking curriculum to include all children, so in partnership with the YMCA, substantial changes were made to the classes. The collaborative modification of the recipes and curriculum between research and YMCA staff was aimed at presenting the information at age-appropriate levels, ensure safety of preparing recipes with numerous children (15+) at a time, and adjust timing to complete in the designated hour allotted. For example, in week 4, to make bruschetta, camp staff and dietitians determined that 7- to 9-year olds would (a) need to use smaller knives and (b) be unlikely to finish a complicated recipe in the allotted hour and that (c) some of the vegetables involved with bruschetta could be off-putting to children. Therefore, for that age group, dietitians and camp staff prepared vegetables for the bruschetta in advance. Each child got to practice dicing a single vegetable, but to save time, the rest were diced by staff before class. Children then mixed the vegetables together and topped the bread. For children not wanting to try the vegetables, they could prepare a simple pesto recipe. This iterative process was beneficial in initial curriculum development, week-to-week modifications, and troubleshooting for future iterations (Table 1). The cooking classes were led by two medical students who underwent training by Brenner FIT staff.

**Table 1:** Cooking camp curriculum

Week	Topic	Recipe	Teaching focus
1	Knife skills and safety	Black bean garden salsa	How to safely hold a knife and use it
			Introduction to the balanced plate
2	Stove-top cooking	Chicken pasta	Introduction to safely using a stove top
			Introduction to different types of pots and pans and their various uses
			Discussion of balanced meals as a single combined plate
3	Vegetables	Roasted broccoli and asparagus, caramelized Brussel sprouts, sautéed asparagus	Focus was to cook with and eat vegetables
			Expanded discussion of vegetables as a part of the balanced plate
			Introduction to safely using an oven
			Utilizing stove-top skills
4	Snacks (for mini meals)	Fresh pesto and bruschetta	Composition of a healthy snack
			Discussion of the satiety and energy benefits of including two different food groups from the balanced plate
5	Breakfast	Egg-wiches on English muffins	Composition of a balanced breakfast, with at least 3 portions of the balanced plate
			Creating different breakfast combinations
6	Lunch	Greek pasta salad and a BLT (bacon-lettuce-tomato) chicken pasta salad	Lunch in relation to the balanced plate
			Discussion on how there is more to lunch than sandwiches
7	Dinner	Pizza bagels and Caprese salad	Creation of a balanced dinner
			Creativity in creating and presenting dishes

Cooking lessons were broken up into groups based on age range. Twice per day, a group of 10 to 15 children would have a 10-min lesson on a given recipe. Cooking camp leaders (medical students) pointed out foods that were likely new and discussed how the ingredients fit into the balanced plate. Children then were divided into groups of three or four to make the recipe. Staff would assist in preparation for younger groups, and let older groups create the recipe with minimal assistance. Once the recipe was completed, staff and campers would “plate” the dish and then eat it at the table with other campers. Staff and leaders refrained from encouraging children to finish or even taste the food, but would simply set it in front of them and let them decide. While they ate, leaders reviewed key aspects of the recipe that were healthy and how it fits into the balanced plate.

YMCA staff regularly communicated with parents of children about the cooking classes through handouts and information sent home with children and weekly email newsletters. Recipes (Additional file 1), educational handouts (Additional file 2), and online videos were extended to parents periodically throughout the summer by all of these means. The cooking camp was held for 7 weeks due to staff availability, focusing on the highest-attended weeks of the summer.

Initially, 10 parents and 10 children were interviewed. Study team members discussed the initial interviews and findings as a group but did not do a formal analysis. It was determined that new information was still being gathered, so an additional 5 interviews were conducted, at which time it was determined that the saturation of responses had been reached.

### Setting

The YMCA of Northwest North Carolina is a 14-branch organization across the Piedmont region of Western North Carolina. The YMCA included in this study houses an instructional kitchen, which is a joint project between the YMCA and Brenner Children’s Hospital (part of Wake Forest Baptist Medical Center). The YMCA day camp is an annual 10-week day camp from 7 a.m. to 6 p.m. Campers can attend any or all of the 10 weeks. YMCA summer camp is open to children ages 5–15 years and engaged in activities ranging from swimming and tag to board games and silent reading. Camp costs range from $100–$150 per week depending on camper age; financial assistance is available for many participants.

### Interview guide

Interview guides were developed for parent and child interviews (Tables 2 and 3). Interviews were designed and conducted using a phenomenological approach to understand children’s and parents’ experience of the cooking camp. Parent interviews were to capture their perception of the child’s experience in the cooking camp, the extent the child discussed the experience at home, and changes in cooking and eating behavior. Child interviews were similarly structured, with additional questions qualitatively exploring their understanding and learning of key nutritional topics taught in the classes: balanced plate, breakfast, snacks, sugar-free drinks, and trying new foods. This method of capturing learning was chosen due to there being few validated nutrition knowledge measures, the wide age range of child participants, and the exploratory nature of this study. Interviews were tested initially for clarity and comprehension via cognitive interviews [35, 36] with volunteers not involved in the study. Cognitive interviewing is a method to develop and refine survey questions by having test subjects read a question, then “think-aloud,” describing their understanding of the question and its implied meaning, as well as describing their thought process in answering the questions. This helps to align the intent of a question with the subjects’ comprehension and understanding of it, allowing for modification and refinement of the question over time. The interview guide was then reviewed for face-validity by dietitians, parents, and child volunteers to determine if questions sufficiently explored the child’s experience and the parent’s impressions of the child’s experience in the camp [37]. Interviews were semi-structured to allow staff to probe for detail, provide clarification, and allow new areas of inquiry to emerge. Interviews with children lasted approximately 5–15 min; interviews with adults lasted 10–15 min.

**Table 2:** Parent interviews

Questions	Response summaries
Has your child talked about the cooking class? What has your child said?	All parents said their child talked frequently about cooking classes.
	Children spoke about recipes made in camp and how much they enjoyed learning kitchen skills, particularly holding and using knives.
Does your child enjoy the cooking classes? What do they like most?	Children thoroughly enjoyed the cooking camp.
	Favorite part was preparing meals and learning new kitchen skills (use of knives, stove-top cooking).
Tell me how your child helps cooking at home.	Most children helped with cooking in the kitchen at home prior to the class.
	Children completed tasks like measuring, chopping, baking, washing, preparing, and assisting with stove-top cooking.
Have you and your child tried any of the recipes from camp at home? Which ones?	Four out of 15 families had tried the recipes at home, with bruschetta being the most common recipe attempted. Five parents said they wanted the recipes but their kids had not brought them home.
Has your child wanted to cook more at home?	Nearly all parents reported their children were wanting to cook more meals at home as a result of the camp.
What has your child learned in the cooking camp?	Most parents reported their children learned safety in the kitchen, like how to cut vegetables.
	Most said their children were eating new and more vegetables and were picking fewer foods out of their dinner.
Do you think you have been cooking differently at home since your child started the camp?	No parent said that they were cooking differently at home since the beginning of camp.
Have you noticed a change in your child’s eating?	Most parents did not notice a significant difference in their child’s eating habits, aside from eating new and more vegetables.
Is your child trying new foods or less picky?	Most parents reported their children were more open and adventurous in trying new foods.
Would you and your child be interested in participating in cooking classes together?	Parents said that they would be very interested in participating in cooking classes together.
Are there any new recipes or kitchen skills that you or your child would like to learn?	Most parents reported wanting to learn healthy alternatives to the food they already cook.
	Parents wanted their children to develop more cooking and kitchen skills.

**Table 3:** Child interviews

Question	Response summaries
What do you like most about cooking camp?	Most children said they like being able to eat what they make.
	Some children brought up skills they’ve learned, such as cutting and using the stove.
	One child said that his mother does not let him cook at home, so he enjoyed getting to see what went into his food.
What is your least favorite thing about cooking camp?	The vast majority of children answered “nothing”
	One child said sharp knives because they scare her.
What was your favorite recipe?	The three favorite recipes were bruschetta, salsa, and chicken pasta
What else would you like to do during cooking camp?	The most common answers were how to make healthy desserts and pizza.

### Analysis

Interviews were analyzed using content analysis [38], which uses a systematic method for coding and analyzing qualitative data to identify meaningful content and giving context to the responses. In particular, content analysis allows for some quantitative reporting of responses. This method was chosen to explore explicit and covert meanings in child and parent interviews, and identify aspects for interpretation, though for this study, this approach was used to summarize responses across participant groups. The systematic coding schema is based on approaches outlined by Bernard and Ryan [38]: first, two investigators (LW, AM) developed a common coding library by separately reading and re-reading transcripts, identifying potential codes. Transcripts and codes were re-reviewed with a third investigator (JAS) to refine the common coding library. Codes were modified as needed by constant comparison and revision. Once the common coding library was developed, all transcripts were analyzed and initially coded by the original two investigators (LW, AM), then reviewed by the third (JAS). Discrepancies in coding were resolved through general discussion, with the third investigator (JAS) adjudicating as needed. For interviews focused on participant experience, responses were summarized by question; for the substantive learning by child participants, they were grouped by learning domains (balanced plate, breakfast, snacks, drinks, healthy foods, new foods).

### Ethical approval

The Institutional Review Board of Wake Forest School of Medicine approved this study (IRB #00038617). For this study, because no health or identifying information was gathered, we used a waived-written consent process. This passive consent informed parents about the study and allowed them to opt out of their children participating if they wished. Parents and children provided verbal consent to participate in interviews. Socio-demographic data were provided in aggregated form by the YMCA and could not be linked to any research data.

## Results

Summer day camp lasted 10 weeks, with the cooking curriculum occurring during the first 7 weeks; no parent or child refused to participate in interviews, and none opted for their child not to participate in interviews or in cooking classes. Eleven of the 15 parents were female/mothers; eight of the 15 children were female, mean age of 9.7 years (range 7–15). From interviews with the children and parents, it emerged that children overwhelmingly enjoyed trying new recipes and attending the cooking camp (Tables 2 and 3). Very few had specific complaints about any aspect of the cooking camp. The favorite recipe for children was bruschetta. For parents, four out of 15 parents reported having already tried one or more of the camp recipes at home, and nine reported planning on trying them. Only two of the 15 families said they had no plans to make any recipes. Most parents reported their children asked to help cook more often at home after attending cooking classes. The most common lesson parents reported their children had learned was about tasting new vegetables. Many parents expressed interest in attending cooking classes with their children.

In exploring nutritional topics with the children, most had a good understanding of the five food groups of the balanced plate and could replicate it with minimal mistakes (Table 4). They named a wide variety of fruits and vegetables they tried and enjoyed when prompted. All identified water as healthy, but many include juice and diet sodas in the healthy category as well. Fourteen out of 15 campers said they enjoyed trying new foods as a result of the cooking camp.

**Table 4:** Child learning interviews

Domains	Themes
Cooking camp experience	Children enjoyed cooking, trying new foods, and cooking with friends.
	Enjoyed being in the kitchen
Nutrition topics	Most children correctly named the 5 major food groups (fruit, vegetables, protein, starch/grains, dairy).
	Children correctly identified pasta, breads, and rice as grains. They noted whole grains as being healthy. They did not mention starchy vegetables or corn as nutritionally similar to grains.
	Most correctly identified milk, cheese, and yogurt as dairy, with several incorrectly identifying soup, bread, cake, and eggs.
	Children correctly identified meats as proteins, also noting nuts, protein shakes, and protein bars.
	Children named a wide variety of fruits and vegetables.
Balanced plate	Children recognize the healthy nature of the balanced plate and the role of the different foods in health.
	Most believed the balanced plate is for daily use.
	When tasked with filling in a blank balanced plate, not all of the details were correct but many children understood concepts.
	Almost all children included dairy on the balanced plate despite dairy not being emphasized in cooking class.
Breakfast	“Gets you ready for the day” was the most popular response for breakfast was important.
	Children mostly identified traditional breakfast foods as being healthy, with most correctly identifying fruits as the healthiest breakfast, but many noting eggs as well.
Snacks	Fruit was the most common food children ate for snacks, followed by crackers and chips.
	Children understood the importance of snacks, noting their value in providing “fuel” in between meals.
	Fruit was by far considered the healthiest snack, followed by vegetables. Many children also named combinations of peanut butter with various fruits.
	Chips were most commonly identified as an unhealthy snack, followed by sweets and “greasy” foods.
Beverages	Almost all children identified water as a healthy drink. Orange juice and milk were also considered healthy. A few children identified diet soda as healthy.
	Most children identified sugar as the ingredient causing a drink to be unhealthy. Others noted “chemicals,” “sodium,” and “calories.”
	Soda was the most common unhealthy drink named.
Foods to choose more often	Many children advocated for the importance of combining food groups from the balanced plate to create a healthy meal.
	Healthy food most commonly meant “being good for your body.” Many children also believed that the number of calories was an important factor.
New foods	14 out of 15 children claimed they try new foods. New foods they tried included Brussel sprouts, spinach, asparagus, pasta, and bruschetta; all of which were ingredients in our recipes.
	Children who enjoyed trying new foods explained that doing so can provide new healthy food options, keeping them from getting tired of the old ones.
	Children ranged from feeling “confident” to “nervous” about trying new foods.

There were no injuries or accidents during the 7 weeks of the cooking camp. The planning of the camp, as well as the research component, required regular and clear communication between YMCA and cooking camp staff, which allowed early identification of allergies or other issues that could negatively impact a child’s experience in the kitchen. One child said they were afraid of sharp knives, which was useful information to relay between cooking and camp staff to prevent situations that could traumatize younger children in the kitchen.

## Discussion

In this study, we explored the feasibility of implementing a cooking curriculum in the YMCA summer day camp; overall, the cooking classes fit well into the schedule and were well received by the child participants. Children were overwhelmingly positive and receptive to the lessons and were excited for the opportunity to cook and eat in the middle of the day. We also preliminarily investigated if children, after participating in the cooking class, could be agents of change in the home cooking environment. Children and parents reported use of the recipes in the home and discussion of the cooking camp sessions at home and noted some positive changes in children’s interest in cooking and receptivity to new vegetables. In teaching children recipes and providing a copy of those recipes to the parents, both children and parents can feel involved and enthusiastic while they create new, healthier habits. These findings are encouraging that a summer day camp cooking curriculum could spur changes in the home meal environment; in line with a few other studies [28, 29], there may be potential to using the child as an agent of change in home meal preparation.

While these preliminary findings are interesting, there are many limitations to this study. First, there are barriers to the expansion of the cooking camp, since many YMCA facilities do not have access to a teaching kitchen. However, many of our recipes only required basic kitchen utensils, so other cooking camps could choose recipes that require only basic items. Because we only conducted interviews once during the summer camp, it is unknown if any families continued to make the new recipes long term. Within the home, we did not capture changes in family meal frequency, or in parent/family nutrition changes. Our sample size was too small to determine if other variables (age, sex, family characteristics) impacted cooking camp experience and extension into the home environment. There may have been bias in the children and parents who volunteered to be interviewed and may not be representative of the entire camp population. Future studies could evaluate these measures with longer and more detailed follow-up. Additionally, future endeavors could attempt implementation on a larger scale (such as a school) to better determine the effects of the cooking classes.

Qualitative interviews revealed that children and parents alike enjoyed the addition of a cooking class into the summer camp. The curriculum developed, and how it was implemented in partnership with a community organization, could be beneficial to others working in this arena. In the effort to increase the number of family meals and decrease the number of meals eaten away from home, using settings in which children spend time may be a potential area of intervention. Children learning to prepare healthy and pleasing meals in a school, day care, or camp setting and encouraged to replicate at home could improve the nutritional practices of families. More work should be done to determine the long-term effects of children as agents of change and how it could be used in conjunction with parent- and family-focused interventions; this approach is a promising avenue for creating change within the family unit and ultimately improving the health habits of children.

## Conclusions

This pilot study demonstrates that cooking lessons can be feasibly implemented into a summer day camp, and children could be agents of change within the home meal environment. Engaging with the YMCA in study planning and execution was key to this implementation. Teaching children cooking skills is a potential area for investigators to impact home meals and nutrition environment, utilizing a community-based setting common to many communities.

## Supplementary information

**Additional file 1.** Pesto Chicken Wrap.

**Additional file 2.** Packing a Balanced Lunch.

## Data Availability

The datasets during and/or analyzed during the current study available from the corresponding author on reasonable request.
