# Supplementary material for: A pilot summer day camp cooking curriculum to influence family meals
Source: Pilot Feasibility Stud. 2019 Dec 13;5:147. doi: 10.1186/s40814-019-0528-0 (PMC6911285; doi:10.1186/s40814-019-0528-0)
Supplement: Supplementary file 1 — Additional file 1. Pesto Chicken Wrap. [file 40814_2019_528_MOESM1_ESM.pdf]

Pesto Chicken Wrap

20 minutes | 6 wraps

Ingredients:

- 1 pound pre-cooked grilled chicken strips
- 1 tablespoon olive oil
- 6 Lite Flat Out Italian wraps
- 6 tablespoons pre-made pesto
- 2 cups spinach
- 4 roma tomatoes, thinly sliced
- 1/2 red onion, finely chopped
- Shredded part-skim mozzarella cheese

Directions:

1. Turn a large skillet to medium-high. Add 1 tablespoon olive oil to the skillet.
2. Place chicken in skillet and cook. Remove chicken from skillet when warm throughout.
3. While chicken is cooking, lay wraps on a flat surface and spread 1 tablespoon of pesto on one long end of each wrap.
4. Place desired amount of spinach and tomatoes on top of pesto.
5. Divide the chicken into 6 equal servings and place on top of the spinach and tomatoes for each wrap.
6. Sprinkle desired amounts of mozzarella cheese and onions on top of chicken.
7. Roll the wrap up by starting with the ingredient end first. Enjoy!
