# Supplementary material for: A pilot summer day camp cooking curriculum to influence family meals
Source: Pilot Feasibility Stud. 2019 Dec 13;5:147. doi: 10.1186/s40814-019-0528-0 (PMC6911285; doi:10.1186/s40814-019-0528-0)
Supplement: Supplementary file 2 — Additional file 2. Packing a Balanced Lunch. [file 40814_2019_528_MOESM2_ESM.pdf]

Wake Forest® Baptist Health
Brenner Children's Hospital

Packing a Balanced Lunch

Aim for 3-4 food groups!

Grains:
Crackers
Sliced Bread
Pita Bread
Tortillas or Wraps
English Muffins
Dry Cereal
Pretzels
Baked Chips
Granola bars

Vegetables:
Celery
Sugar Snap Peas
Sliced Bell Peppers
Baby carrots
Broccoli
Cauliflower
Grape Tomatoes
Spinach
Lettuce

Meat/Meat Substitutes:
Lean deli meat
Nuts: walnuts, peanuts, cashews, etc
Nut butters
Beans and Legumes: pintos, chickpeas, black beans, lentils
Canned meat: chicken, salmon, tuna
Hummus
Pre-grilled chicken pieces
Eggs

Dairy:
Milk
Laughing Cow cheese
Sliced or cubed cheese
String cheese
Yogurt
Eggs

Fruit:
Apple slices
Oranges
Grapes
Raisins, Craisins
Applesauce
Mandarin Oranges
Pears

When you pack 3-4 food groups in your lunch box, you are working towards eating a balanced plate! Having a mixture of 3-4 food groups helps to fill your tummy up and last until the next meal!

Wake Forest® Baptist Health
Brenner Children's Hospital

Balanced Lunch Examples

Peanut Butter and Apple sandwich on Whole Wheat Bread<br>Carrots w/light ranch	Pita Bread Pizza with marinara and Mozzarella Cheese<br>Bell Peppers w/honey mustard<br>Light Yogurt	Whole Wheat Mini Bagel with Cream Cheese & Strawberries<br>Sugar Snap Peas w/light ranch
Turkey and Cheese on a Flat-Out Wrap with lettuce, onions<br>Canned Mandarin Oranges	Pasta Salad with pre-grilled chicken strips, broccoli & carrots<br>Unsweetened Applesauce	Trail Mix with Cereal, Pretzels, Raisins and Craisins<br>Baby Carrots with Hummus
Crackers & Cucumbers with Garlic Hummus<br>String Cheese<br>Fresh Pineapple	Cheese and Spinach Quesadilla<br>Sliced Mango<br>1% Milk	Tuna Salad with Crackers<br>Sliced Celery<br>Grapes

Each balanced lunch box example contains 3-4 food groups!
